# Structural and Immunological Effects of Skin Cryoablation in a Mouse Model

**DOI:** 10.1371/journal.pone.0123906

**Published:** 2015-03-30

**Authors:** Akira Kasuya, Isao Ohta, Yoshiki Tokura

**Affiliations:** 1 Department of Dermatology, Hamamatsu University School of Medicine, Hamamatsu, Japan; 2 Ultrastructural Morphology Laboratory, Research Equipment Centre, Hamamatsu University School of Medicine, Hamamatsu, Japan; University of Bergen, NORWAY

## Abstract

Cryoablation is therapeutically applied for various disorders in several organs, and skin diseases are typical targets as this cryotherapy has been widely used for viral warts, benign tumors, and actinic keratosis. The main mechanisms of cryoablation consist of direct freezing effect on skin constituents, thrombosis formation in microcirculation, and subsequent immunological responses. Among them, however, the immunological mechanism remains unelucidated, and it is an issue how the direct freezing injury induces immunological consequences. We established a mouse cryoablation model with liquid nitrogen applied to the shaved back skin, and used this system to study the immunological excitement. After application of liquid nitrogen, the thermal decrease ratio was -25°C/sec or less and the lowest temperature was less than -100°C, which was sufficient to induce ulceration. Destruction of cornified layer and necrosis of epidermal cells were observed in transmission electron microscopy image, and increased transepidermal water loss and skin permeability were detected by the functional measurements. By flow cytometry, antigen-presenting dendritic cells (DCs), including PDCA1^+^B220^+^CD19^-^ plasmacytoid DCs (pDCs) and CD11c^+^ myeloid DCs, as well as neutrophils and macrophages were increased in subcutaneous tissue. In parallel, the mRNA expressions of interferon α1 which are known as pDC-producing cytokines, was elevated. We also found marked degranulation of mast cells, providing a possibility that released histamine attracts pDCs. Finally, FITC migration assay revealed that pDCs and CD11c^+^ DCs emigrated from the cryoablated skin to the draining lymph nodes. Our study suggests that cryoablation induces destruction of the barrier/epidermis, accumulation of pDCs and CD11c^+^ DCs to the skin, and migration of DCs to regional lymph nodes. Viral elements or tumor cell lysates released from damaged keratinocytes may stimulate the DCs, thereby leading to antiviral or antitumor effect.

## Introduction

Cryoablation is therapeutically applied for various disorders in several organs. Even internal malignancies are currently treated with cryoablation as alternative therapy for surgical resection[1]. Cryoablation is frequently used in daily dermatological clinics for the treatment of human papillomavirus (HPV) warts and skin tumors. Liquid nitrogen (LN_2_) at a temperature of -196°C is a usually employed coolant. LN_2_ is applied easily by means of a cotton wool swab, which is a minimally invasive, inexpensive and simple method.

The main mechanisms of cryoablation consist of direct freezing effect on skin constituents, microthrombosis formation[2], and immunological responses[3]. The direct freezing injury gives rise to varying degrees of epidermal and dermal necrosis. The cold tolerance ability is different among individual skin constituent cells. For example, temperature about -20°C to -30°C is necessary for the death of keratinocyte, while fibroblasts die at -30°C to -35°C[4]. Temperature at least bellow -40°C is required for the necrosis of malignant tissues[5, 6]. Very rapid freezing rate is also essential for cellular death[7]. Rapidly formed intracellular ice is expected to destruct cell membrane as well as cell organelles, such as mitochondria and endoplasmic reticulum. Meanwhile, the extracellular ice changes osmotic gradients between cytoplasm and extracellular fluid, which causes irreversible cell damage and death. Even in the melting phase, intracellular recrystallization of water induces tissue destruction. The thawing rate should be as slow as possible to maximize the recrystallization. Necrosis of vascular endothelial cells produces the thrombosis of capillaries, shutting down the blood flow.

Compared to the direct and the capillary damages, the immunological effect of cryoablation is not clear. Dermatologists occasionally experience the regression of all verruca lesions after cryoablation to even a few lesions, suggesting that the cryoablation induces an immunological response to warts. Furthermore, metastatic foci may regress after ablation of a primary tumor[3, 8–10]. These observations support the existence of immunological effect of cryoablation. However, the mechanism underlying this therapy remains poorly elucidated, and only limited information on infiltrating cells and cytokine profile is available.

It seems that the immunological cascade in cryoablation is initiated by the necrosis of skin constituents. The intracellular ice is expected to destruct cell membrane as well as cell organelles[5, 11]. The disrupted cells spill intracellular contents, such as RNA, DNA, uric acid, HSP70 and chromosomal protein, which might function as damage-associated molecular patterns (DAMPs)[1, 12, 13] or pathogen-associated molecular patterns (PAMPs) when viral infection exists. These substances are thought to provoke infiltration of leukocytes and dendritic cells (DCs) through stimulation of inflammasome or toll-like receptors (TLRs). Subsequently, cytokines released from these cells and cytotoxic T cells (CTLs) primed by virus or tumor-associated antigens might attack tumor cells and HPV-infected cells[14].

We thus hypothesized that cryoablation induces the sequential events where innate immune molecules and DCs are involved. Acquired immunity also may follow them, because the recognition of the cell-derived antigens by cutaneous antigen-presenting cells (APCs) may result in the systemic immune responses. Here, we established a cryoablation mouse model. By using this system, we investigated in the direct damage of skin tissues and the subsequent immunological consequences of cryoablation, focusing on DCs.

## Method

### Animals

Balb/c wild-type mice were obtained from SLC Inc. (Hamamatsu, Japan). All mice were healthy, fertile, and did not display any evidence of infection or disease. Female mice (8- to 12-week-old) were used for all the experiments. All mice were housed in specific pathogen-free barrier facility and screened regularly for pathogens and were held under standard conditions (12 h light: 12 h dark photoperiod cycle, temperature 23±2°C). Mice were individually housed in plastic cages to prevent tampering with the resultant ulcer by other mice. Food and tap water were available ad libitum. For sampling of tissues, mice were anesthetized with diethyl ether and sacrificed by cervical dislocation. All studies and procedures were approved by the Committee on Animal Experimentation of Hamamatsu University School of Medicine.

### Cryoablation

**Fig 1:**
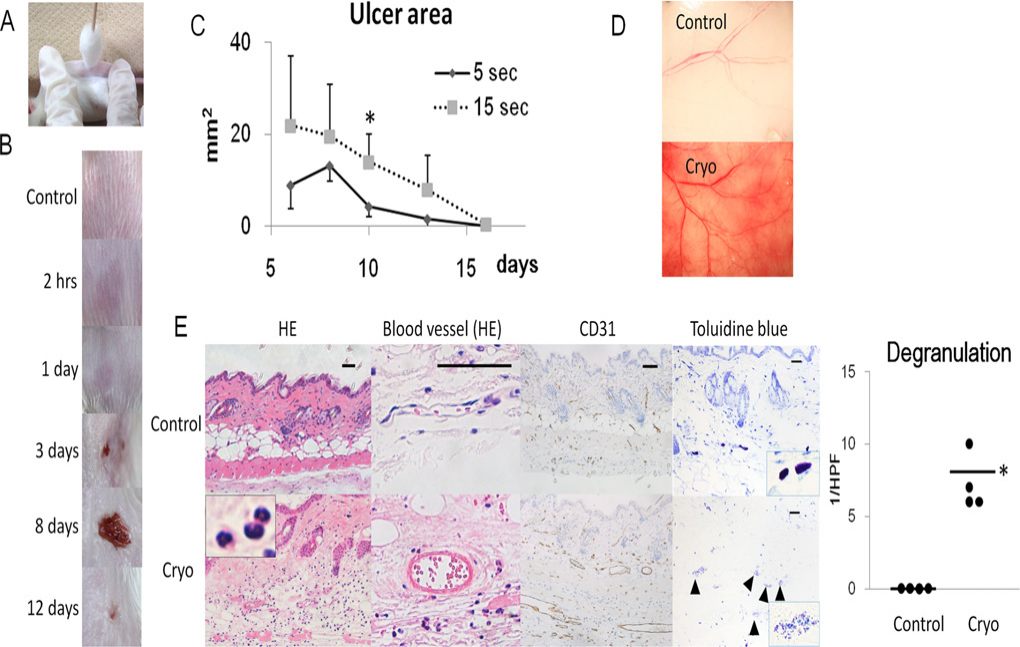
Cryoablation mouse model. A) Cryoablation technique using cotton swab. B) Macroscopic changes of the back after 15-sec cryoablation. C) Chronological changes of ulcer size of the back skin. The formed ulcer is larger in the 15-sec cryoablated group than in the 5-sec ablated group. D) Macrosopical changes of subcutaneous blood vessels in the 15-sec cryoablated group at day 1. E) HE stain, CD31 immunostaining (blood vessel marker), and truidine blue staining of the 15-sec cryoablated skin at day 1. Square box in HE stain shows a high power image of infiltrating cells, mainly neutrophils. The number of degranuated cells is also presented. Square box in toluidine blue stain shows high power image of degranuated mast cells (▲; arrowhead). All scale bars, 20 μm. Cryo; cryoablation. sec; seconds. HPF; high power field. Values represent the mean ± SEM (C). Data are shown as dots (E). Bars represent the mean values. Significant differences between sample means are indicated, *P<0.05 versus 5 sec in (C) and control in (E). n = 4, in each group.

Mice were anesthetized with diethyl ether and their backs were shaved and cleaned with 70% ethanol. The skin was gently pulled up, and LN_2_-soaked cotton swab with a pointed end, which did not exceed 5 mm, was applied to the skin with pressure of 30 gW (Fig 1a). The applying time was 5 sec or 15 sec. A cycle of cryoablation was performed in each mouse. The mice were randomly assigned into individual groups. The skin tissues were harvested and used for the subsequent analysis at 2 hrs or 1 day after cryoablation. The macroscopical symptoms including purpura and ulcer were checked every day until day 14 in each mouse. The ulcer diameter was measured by digital caliper (As one corporation, Osaka, Japan). The largeness of ulcer was calculated as (short diameter)× (long diameter)/2.

### Histological examination and immunohistochemistry

After the mice were sacrificed, cryoablation applied areas were harvested. The samples were cut into halves, fixed in 3.5% paraformaldehyde, and embedded in paraffin. Sections were stained with hematoxylin & eosin (HE), and toluidine blue. Immunohistochemistry was performed using polyclonal rabbit antibody specific for CD31 (Thermo Fisher Scientific Inc., Waltham, MA). Histofine Simple Stain MAX-PO (Nichirei Co., Tokyo, Japan) was used for second antibody. The other half part of the wound was embedded in O.C.T. Compound (Sakura Fine Tech Japan, Tokyo, Japan). For immunohistochemistry, fresh frozen sections were fixed with 100% methanol and treated with 3% normal chicken serum (Gibco; Life Technologies Corporation, Carlsbad, CA) for 6 minutes at room temperature. Sections were then incubated with primary antibodies, which were the polyclonal rabbit antibody for Zo-1, the monoclonal mouse antibody specific for PDCA1 (Biolegend, San Diego, CA) and E-cadherin (Takara Co., Tokyo, Japan). Alexa Fluor Secondary Detection Reagents (Alexa Fluor 555 donkey anti-rabbit IgG (H+L) antibody for Zo-1, Alexa Fluor 555 goat anti-mouse IgG (H+L) for PDCA-1, Alexa Fluor 488 rabbit anti-mouse IgG (H+L) for E-cadherin; Life Technologies Co., Carlsbad, CA) were used as secondary antibody. The stained sections were observed by a fluorescence microscope (BX51, Olympus, Tokyo, Japan).

### Detection of Temperature of Skin surface and Subcutaneous tissue

Digital thermometer (ERA-2000K, Ando Keiki, Tokyo, Japan) was used for detecting temperature. Probe was inserted to the subcutaneous tissue beneath the skin area of cryoablation. The skin surface temperature was monitored by thermography camera (TVS-100, Nippon Avionics Co., Ltd Tokyo, Japan).

### Transmission electron microscope (TEM)

The observation by TEM was performed as previously reported[15]. The skin sample was fixed with 2.0% glutaraldehyde in 0.1 M phosphate buffer (pH 7.4) for 2 h at 4°C, washed three times in the same buffer and fixed with 1% osmium tetroxide in 0.1 M phosphate buffer for 2 h at room temperature as previously delineated[15]. The skin was then washed in buffer, dehydrated in graded ethanol, and embedded in Epon. Ultrathin sections (60 to 80 nm thick) were cut using a Reichert ultramicrotome OmU4, collected on copper grids, double-stained with 2% aqueous uranyl acetate and lead citrate and observed in a JEM-1220 (JEOL Ltd., Japan) transmission electron microscope under 80 kV.

### Detection of transepidermal water loss (TEWL) and corneal water content

Mobile Tewameter (TM300MP, Integral, Tokyo, Japan) was used for the measurement of TEWL at cryoablated area. Corneal water content at cryoablated area was measured by corneal water content meter (SR-101, LOZENSTAR, Kawasaki, Japan).

### Lucifer yellow, permeability assay for measurement of skin barrier

Lucifer yellow (Sigma-Aldrich, St. Louis, MO) was diluted in PBS and added to the apical compartment at a final concentration of 100 μM. Fifty μL of lucifer yellow dissolved in PBS was put on the sampled skin surface. The skin specimen was incubated in a 37°C incubator (5% CO_2_ and 90% humidity) for 2 hrs and washed with PBS. Frozen section was observed with a fluorescence microscope (BX51, Olympus, Tokyo, Japan). The depth of permeated lucifer yellow was measured.

### Real-time PCR

Total RNAs were extracted from skin samples using QIAGEN RNeasy spin columns (QIAGEN Ltd., Crawley, UK) and digested with DNase I (QIAGEN Ltd.) to remove chromosomal DNA in accordance with the manufacturer’s protocols. Total RNA was reverse transcribed to cDNA using a reverse transcription system with random hexamers (Promega, Madison, WI). Real-time PCR was performed in triplicate using the TaqMan gene expression assays (Applied Biosystems, Foster City, CA) on an ABI Prism 7000 Sequence Detector (Applied Biosystems) according to the manufacturer’s instructions. TaqMan probes and primers for interferon (IFN)-α1, IFN-γ, retinoic acid receptor responder-2 (RARRES2; chemerin), tumor nocrosis factor (TNF)-α, monocyte chemotactic and activating factor-1 (MCP-1), and glyceraldehyde-3-phosphate dehydrogenase (GAPDH) were purchased from Applied Biosystems. Relative expression of real-time PCR products was determined using the ΔΔCt technique[16]. Briefly, each set of samples was normalized using the difference in threshold cycle (Ct) between the target gene and housekeeping gene: ΔCt. (Ct target gene-Ct GAPDH). Relative ratio of mRNA levels was calculated as 2^-ΔΔCt^, where ΔΔCt = ΔCt sample-ΔCt calibrator. The smallest ΔCt value in control data of each gene was used as the calibrator[16]. Using the ΔΔCt method, the data are presented as the fold change in gene expression normalized to an endogenous reference gene and relative to the control data. For the control sample used as calibrator, ΔΔCt equals zero, and 20 equals one, so that the fold change in gene expression relative to the control equals one, by definition. The number of mice used was 4 in each group.

### Flow Cytometric Analysis

Skin-infiltrating inflammatory cells were isolated and subjected to flow cytometric analysis. Cryoablation-treated back skin with the largeness of 3cm^2^ was harvested. Subcutaneous tissue was peeled by scalpel and was minced with the surface of frosted glass in phosphate-buffered saline (PBS, pH 7.4). Then, the tissue was washed with PBS. The fluid was filtered through a 70-μm nylon mesh to obtain a single-cell suspension.

Flow cytometric analysis was performed as previously suggested[17]. The following primary antibodies were used: phycoerythrin (PE)-labeled anti-mouse B220 monoclonal antibodies (Clone: RA3-6B2; Biolegend, San Diego, CA), allophycocyanin (APC)-labeled anti-mouse PDCA1 monoclonal antibodies (Clone: 927; Biolegend), PE-labeled anti-mouse CD11c monoclonal antibodies (Clone: HL3; BD Pharmingen, San Diego, CA), APC-labeled anti-mouse CD11b (Clone: M1/70, BD Pharmingen), PE-labeled anti-mouse CD49b (Clone: HMα2, Biolegend), fluorescein isothiocyanate (FITC)-labeled anti-mouse TCR γ/δ (Clone: Biolegend), FITC-labeled anti-mouse CD3 (Clone: GL3, Biolegend), (FITC)-labeled anti-mouse CD19 (Clone: 6D5, Biolegend), APC-labeled anti-mouse CD4 (Clone: RM4-5, Biolegend), PE-labeled anti-mouse CD8 (Clone: 53–6.7, Biolegend). All antibodies were used at 1:200 dilution according to manufacturer’s instructions. Incubation was performed for 15 min at room temperature, followed by two washes in PBS supplemented with 5% fetal calf serum (FCS) and 0.02% sodium azide. 7-Amino-actinomycin D (7-AAD; BD Pharmingen) was added to exclude dead cells, which are 7-AAD+. FACSCanto II (Japan BD, Tokyo, Japan) was used to obtain fluorescent profiles. Data analysis was performed using Flowjo software (Treestar, Inc. Ashland, OR). Gating strategy is as follows. First, SSC-A / FSC-A was used for obtaining monocytes, dendritic cells and lymphocytes. Secondary, FSC-W / FSC-H was used for singlets. Thirdly, we adopted SSC-W / SSC-H for additional gating of singlets. Fourthly, SSC-A / 7-AAD was used to exclude dead cells, which are 7-AAD+. Moreover, for CD19-B220+PDCA1+ cells, SSC-A / CD19 was used.

### Migration assay

PDCA1^+^ pDCs and CD11c^+^ mDCs, migrated to inguinal lymph nodes from the cryoablated area, were analyzed by FITC skin painting method as previously described[18]. In brief, FITC (Sigma-Aldrich, St. Louis, MO) was dissolved (5 mg/ml) in a 50:50 (vol/vol) acetone-dibutylphthalate mixture just before application. The 15-sec cryoablation was performed on the back skin. Then, 0.2 ml of FITC solution was painted on the cryoablated area. Usually, FITC is permeates into skin tissue, binds to proteins, and is taken in by DCs, which migrate into the regional inguinal lymph nodes. After 48 hrs, inguinal lymph nodes were sampled and mechanically minced. Cell suspensions were meshed, washed in PBS, and stained with an APC-labeled anti-mouse PDCA1 monoclonal antibody (Clone: 927; Biolegend) and PE-labeled anti-mouse CD11c monoclonal antibodies (Clone: HL3; BD Pharmingen, San Diego, CA). FITC^+^PDCA1^+^ cells and FITC^+^CD11c^+^ were detected by FACSCanto II (Japan BD, Tokyo, Japan). Data analysis was performed using Flowjo software (Treestar, Inc., Ashland, OR). Gating strategy is as follows. First, SSC-A / FSC-A was used for obtaining monocytes, dendritic cells and lymphocytes. Secondary, FSC-W / FSC-H was used for singlets. Thirdly, we adopted SSC-W / SSC-H for additional gating of singlets. Fourthly, SSC-A / 7-AAD was used to exclude dead cells, which are 7-AAD+.

### Statistical analysis

The two-tailed unpaired Student’s t-test was used to determine the level of significance of differences between the sample means. A p-value <0.05 was considered statistically significant.

## Results

### Establishment of cryoablation model

**Fig 2:**
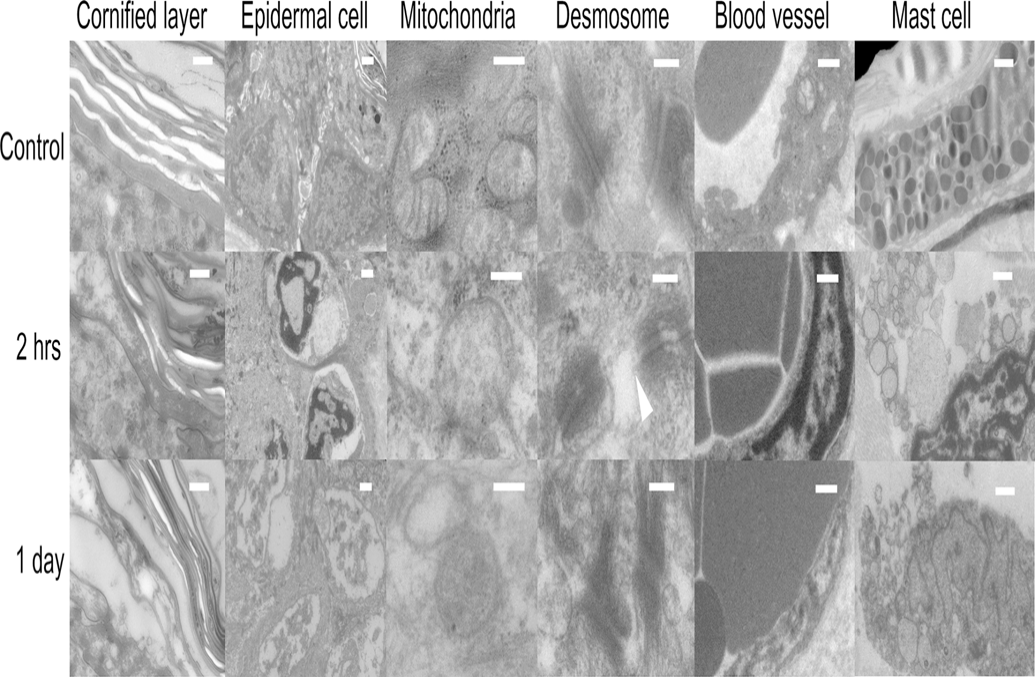
Transmission Electron Microscope (TEM) image of skin constituents. The length of scale bars is 0.5 μm in cornifyed layer, 1 μm in epidermal cells, 0.2 μm in mitochondria, 0.2 μm in desmosomes, 1 μm in blood vessels and 1 μm in mast cells. All constituents were observed at 2 hrs and 1 day after cyoablation. Discontinous cell menbrane (▲, arrowhead) was observed in the vicinity of desmosome, suggesting dysruption of cell menbrane by cryoablation.

Cryoablation of the back skin of Balb/c mice with cotton swab (Fig 1A) evoked vasodilation and purpura immediately after the treatment, followed by necrosis and ulceration (n = 4, Fig 1B and 1C). Congestion and bleeding of subcutaneous blood vessels became congested with bleeding were observed, suggesting the shutdown of blood flow at day 1 (Fig 1D). Blood vessels were dilated with denatured endothelium at day 1 as shown in the Hematoxylin-Eosin (HE) staining and CD31 immunostaining (Fig 1E). Transmission electron microscopy (TEM) showed coagulated red blood cells stuck in the lumen of blood vessel at day 1. The nucleolus of endothelial cell was condensed at 2 hrs after cryoablation and dissolved at day 1, indicating the necrosis (Fig 2).

Toluidine blue staining (n = 4, Fig 1E) and TEM images (Fig 2) revealed degranulation of mast cells at 2 hrs after cryoablation. This could contribute to dilation of blood vessel observed at 2 hrs, because granules of mast cells contain a large amount of histamine, a potent vasodilator[19].

### Temperature change after cryoablation

**Fig 3:**
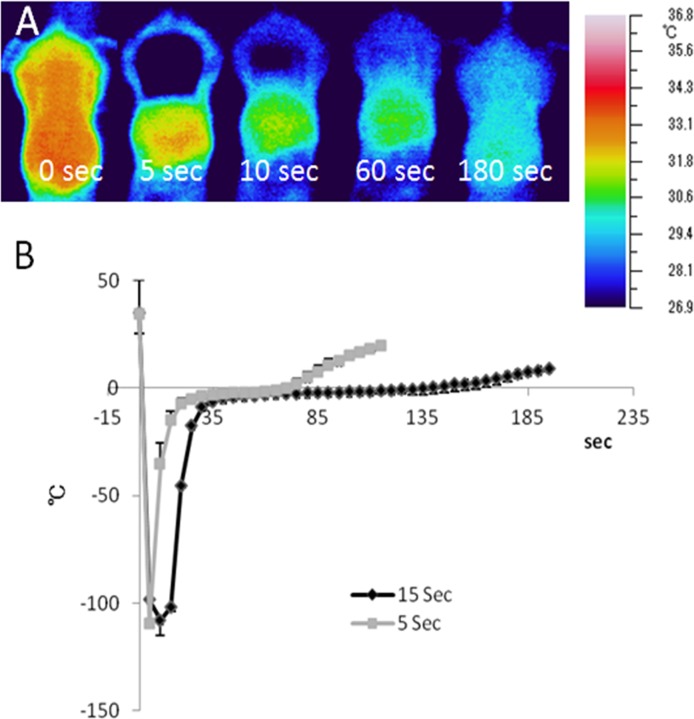
Changes of temparature of tissues after cryoablation. A) The temperature change of skin surface after 5-sec or 15-sec cryoablation was monitored by thermography. The thermometer probe was inserted into the subcutaneous tissue. B) The temparature recovered more slowly in the 15-sec than in the 5-sec cryoablated group. The increase of temparature became blant just bellow 0°C, where recrystallization and the following tissue damage occur. sec; seconds. All values represent the mean ± SEM. n = 4, in each group.

The surface temperature was dropped immediately after application of LN_2_-soaked cotton swab. It recovered at 180 sec after cryoablation (Fig 3A). Cryoablation rapidly decreased the temperature of the subcutaneous tissue bellow -100°C at a rate of -25°C/sec (n = 4, Fig 3B). This decline speed is sufficient to cause severe cell damage[7]. The 15-sec application needed a longer time than the 5-sec application for recovering of temperature above 0°C. The slow increase of temperature induces recrystallization of intracellular water, causing damage to the cell[7].

### Destruction of skin barrier by cryoablation

**Fig 4:**
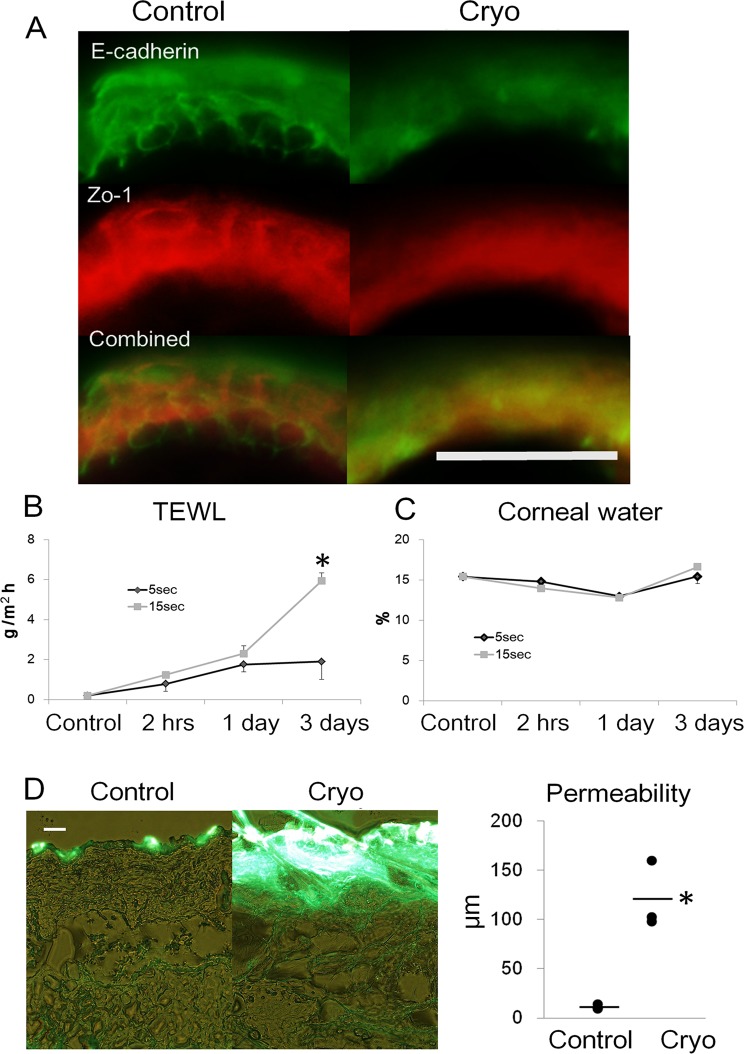
Functional and morphological changes of skin barrier. A) Immunohistochemistry of E-cadherin and Zo-1 after 15-sec cryoablation at day 1. Scale bar, 100 μm. The change of transepidermal water loss (TEWL, B) and corneal water content C). D) Increased skin permeability of lucifer yellow after 15-sec cryoablation at day 1. Green fluorescence shows the permeation of lucifer yellow. Cryo, cryoablation; scale bar, 100 μm; and sec, seconds. Values represent the mean ± SEM (B, C). Data are shown as dots (D). Bars represent the mean values. Significant differences between the means are indicated, *P<0.05 versus 5 sec in (B) and control in (D). n = 4, in each group of (B) and (C). n = 3, in each group of (D).

TEM showed degeneration of cornified layer with increased interlayer spaces at 2 hrs and 1 day after cryoablation (Fig 2). The nucleoli of epidermal cells were condensed at 2 hrs and dissolved at 1 day after cryoablation. The internal structure of mitochondria was blurred, suggesting the destruction of membrane. Desmosome was relatively preserved, but attached membrane was shred and distorted. Immunohistochemistry (IHC) showed that E-cadherin and Zo-1 fringed epidermal keratinocytes in the non-treated skin (Fig 4A). However, the structure was blurred after cryoablation, reflecting the destruction of cell membrane. E-cadherin is a cell attachment apparatus existing on cell membrane[20]. Zo-1 exists mainly on the tight junction which is located on the upper epidermal layer[21]. The disappearance of these structures suggests the damage and distortion of cell membrane.

The barrier function was examined by transepidermal water loss (TEWL) (n = 4, Fig 4B), corneal water content (n = 4, Fig 4C), and permeability assay (n = 3, Fig 4D). TEWL was increased even 2 hrs after cryoablation without any change in corneal water content. Permeability assay showed increased permeability of lucifer yellow. In the healthy control epidermis, lucifer yellow does not permeate through skin barrier, because it has a high molecular weight (MW 457.25)[22]. Thus, the increased permeability of lucifer yellow represented the dysfunction of skin barrier.

### Immunological changes by cryoablation

HE staining showed that the infiltrating cells were mainly neutrophils at 1 day after cryoablation (Fig 1E). Degranuation of mast cells was also observed (n = 4, Fig 1E).

**Fig 5:**
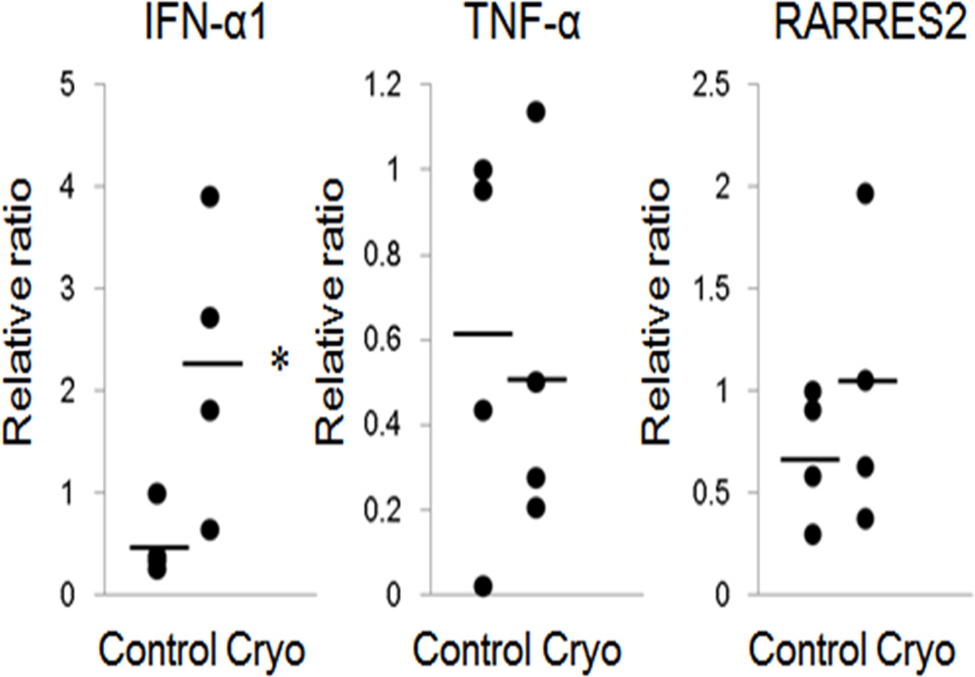
mRNA expression in the skin treated with cryoablation. The mRNA expressions of IFN-α1, TNF-α, and RARRES2 were measured by real time PCR analysis at day 1 in 15-sec cyoablated skin. The levels of TNF-α, and RARRES2 (potent chemoattractant of pDCs), were not increased by cryoablation. Cryo, cryoablation. All data are shown as dots. Bars represent the mean values. Significant differences between the means are indicated, *P<0.05 versus control. n = 4, in each group.

The mRNA expressions of IFN-α1 was elevated at 1 day after cryoablation (n = 4, Fig 5). The mRNA expression of TNF-α, and RARRES2, a strong chemoattractant of pDCs, was not increased by cryoablation. IFN-γ and MCP-1 were under detecting level in control and cryoablated mice (data not shown).

**Fig 6:**
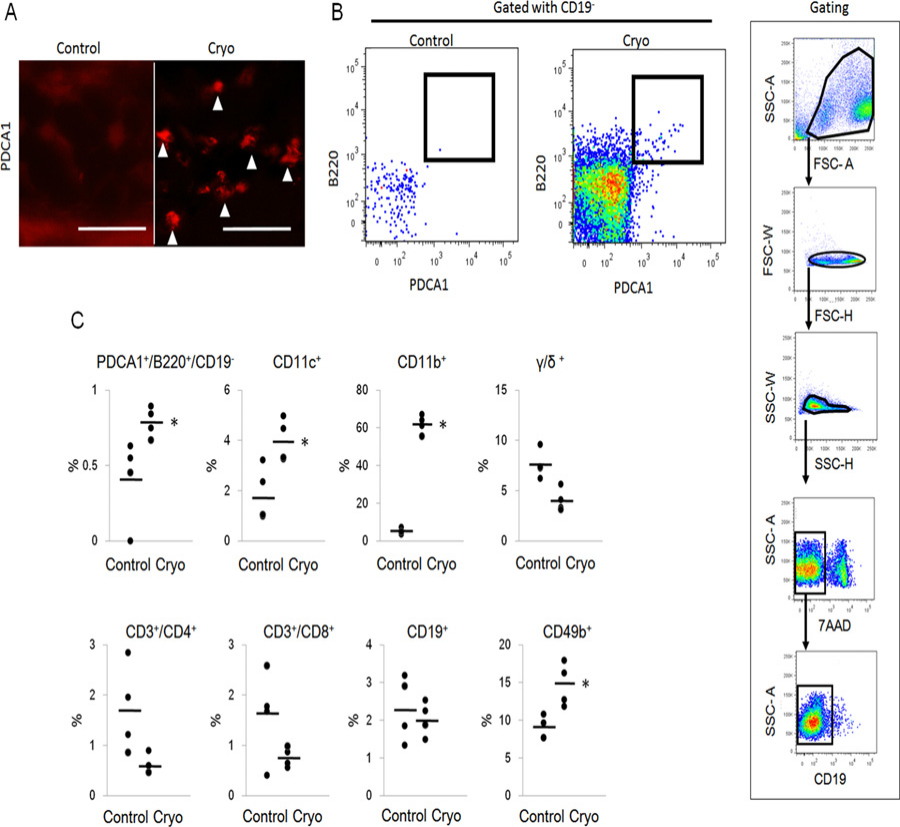
Profile of infiltrating cells. A) Immunohistochemistry of PDCA1. PDCA1^+^ cells infiltrate in the cryoablated skin (▲, arrowhead). Scale bars, 20 μm. B)C) Flow cytometry analysis of infiltrating cells. B) Dots within bold square box shows PDCA1^+^B220^+^CD19^-^ plasmacytoid dendritic cells. Gating strategy is also shown. Dead cells, which are 7-AAD^+^, were removed. We used CD11c for mDCs, CD11b for neutrophil and macrophage, TCR γδ for γδ T cells, and CD49b for natural killer cells. Cryo, cryoablation. All data are shown as dots (C). Bars represent the mean values. Significant differences between the means are indicated, *P<0.05 versus control. n = 4, in each group.

By immunohistochemistry, PDCA1^+^ cells, representing plasmacytoid dendritic cells (pDCs), infiltrated in the subcutaneous tissue of cryoablated skin (Fig 6A). Flow cytometry analysis of infiltrated cells showed increased numbers of PDCA1^+^B220+CD19^-^ pDCs (n = 4, Fig 6B and 6C), CD11c^+^ myeloid dendritic cells (mDCs), CD49b^+^ natural killer cells, and CD11b^+^ cells (neutrophils plus macrophages) (n = 4, Fig 6C). The frequency of CD3^+^CD4^+^ T cells, CD3^+^CD8^+^ T cells, TCR γδ T cells and CD19^+^ B cells were not increased in the cryoablated skin. (n = 4, Fig 6C).

**Fig 7:**
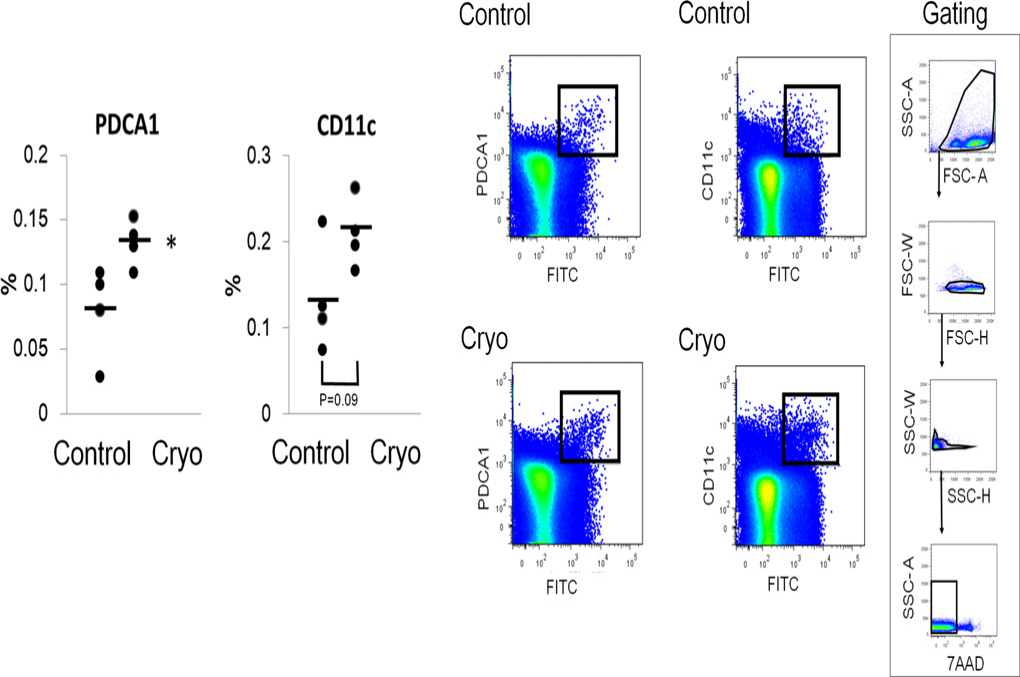
Migration assay of dendritic cells to regional lymph nodes. Flow cytometry analysis for migrated FITC^+^CD11c^+^ myeloid dendritc cells and FITC^+^PDCA1^+^ plasmacytoid dendritc cells. Gating strategy is also shown. Cryo; cryoablation. All data are shown as dots. Bars represent the mean values. Significant differences between the means are indicated, *P<0.05 versus control. n = 4, in each group.

We performed the migration assay to examine whether DCs actively emigrate to the draining lymph nodes by using fluorescein isothiocyanate (FITC) which binds to cells and function as hapten. FITC was applied to the cryoablated or non-treated back skin of the mice, and the regional lymph node cells were sampled to analyze the frequencies of FITC^+^CD11c^+^ DCs and FITC^+^PDCA1^+^. The migration of FITC^+^CD11c^+^ DCs and FITC^+^PDCA1^+^ into the lymph node tended to increase in the cryoablated mice (n = 4, Fig 7). The results suggest that FITC applied to the back skin penetrated into the skin tissue, then CD11c^+^ DCs and pDCs actively migrated to the regional lymph nodes.

## Discussion

In this study, we used a mouse cryoablation model with LN_2_ applied to the shaved back skin. The thermal decrease after application of LN_2_ was -25°C/sec or less, and the lowest temperature was less than -100°C. By both morphological and functional methods, we confirmed the damaged cornified layer and epidermal cell necrosis with increased TEWL and skin permeability. The blurred immunostaining of Zo-1 and E-cadherin indicated the destructed structure of cell membrane. The destructed microcirculation is also important in cryoablation[2]. In our study, the freezing immediately stopped the microcirculation, which was restarted after thawing. The subsequent vasodilatation and increased vascular permeability resulted in severe edema and inflammatory cell infiltration. Injury of capillary endothelial cells caused platelet aggregation, microthrombosis, and blood flow stasis, as observed in the HE staining and CD31 immunostaining at day 1. TEM exhibited the necrosis of endothelial cells. Diverse mechanisms are likely to underlie the vascular change. We found that mast cells were degranulated upon cryoablation. The destruction of cell membrane of mast cell may increase the influx of Ca^++^, which induces the degranulation and histamine release[22], accelerating the capillary hyperpermeability.

Cryoablation triggers systemic immunological responses[3, 8–10]. Our flow cytometric analysis showed the increased infiltrates of PDCA1^+^B220^+^CD19^-^ pDCs and CD11c^+^ mDCs as well as CD11b^+^ neutrophils and macrophages after cryoablation. Accumulation of these cells represents operation of innate immunological responses following cryoablation. DCs consist of normal regional constitutive CD11c^+^ mDCs and PDCA1^+^B220^+^CD19^-^ pDCs. The limit of our study is that PDCA1 is predominantly specific for mouse pDC in naive mice, but is up-regulated on most cell types following stimulation with type I IFNs and IFN-γ[23]. Thus as an marker of pDC, PDCA1 is not enough. Activated pDC produces large amounts of type I IFNs capable of exerting strong anti-virus and anti-tumor effects. We found the increased expression of IFN-α in our cryoablated skin model. However, we could not observe the elevated expression of IFN-γ or TNF-α, which were reported to be increased by cryoablation in a model of kidney tumor [24]. The major limitation is that tumor and virus are absent in our system. The FITC migration assay showed the increased migration of FITC^+^ pDCs and FITC^+^ mDCs to regional lymph nodes, suggesting that these DC populations are activated by DAMPs or PAMPs derived from damaged epidermal cells. The migrated pDCs undergo maturation. The increased migration of DCs to regional lymph nodes may be promoted by cryoablation-induced attenuation of barrier function.

The cryoablation-evoked destruction of *stratum corneum* and epidermis may have a greater impact on the therapeutic efficacy to HPV infection. HPV usually evades from immunological recognition by avoiding the main triggers that initiate an immune response to viral infection[25]. HPV neither destroy the infected cells nor have virus-associated double-stranded RNA to evoke innate immune responses. Furthermore, HPV expresses late proteins only in the upper epithelial granular and cornified layers, where inflammation does not easily occur[26, 27]. However, once HPV is recognized by immune system, verruca lesions can spontaneously regress, as seen in flat warts[28]. Therefore, immunological activation could be a potent strategy to repel HPV infection. Cryoablation destructs the intact skin barrier and bursts cell membrane of infected epidermal cells, exposing the ‘hidden’ virus antigen to the immune system. The exposure of viral late proteins to the immune system might help the regression of existing HPV lesion. Currently, we cannot define the individual roles of pDCs and mDCs in viral or tumor elimination. The primary role of pDCs was initially defined by the production of type I IFNs in response to viral infection, and pDCs have a number of characteristics that differ from conventional DCs. Their antigen-presenting function is often neglected, since most studies on antigen presentation are aimed at other DC subsets. However, recent studies have suggested that pDCs can serve as professional antigen-presenting cells to eliminate tumors[29]. It seems that HPV-infected cells or tumor cells are attacked by type I IFNs released from pDCs and/or CTLs primed by pDCs or other DCs stimulated by virus or tumor-associated antigens. In this scenario, cryoablation may provide antigens to these DCs and stimulate them to become efficient APCs for CTLs.

The mechanism of pDC infiltration in cryoablated skin remains unclear in this study. None of the two chemoattractants for pDCs, which are RARRES2 and MCP-1, was increased by cryoablation. However, mast cell-derived histamine is a candidate for pDC accumulation. Histamine increases the capillary hyperpermeability assisting the infiltration of inflammatory cells into the affected tissue. Histamine even chemoattracts pDCs and monocyte derived DCs through histamine receptor 4 (H_4_)[30, 31]. Histamine receptor 2 (H_2_) enhances monocyte derived DCs by producing matrix metalloproteinase (MMP)[32]. Histamine may contribute to the migration of pDCs and mDCs to lymph nodes[33].

Our study suggests that the cryoablation stimulates DC populations to accumulate in the treated skin and to migrate to the lymph nodes. Thus, this conventional simple cryotherapy rather induces orchestrated immunological consequences. Such physical injuries could be re-evaluated from the viewpoint of recent advances in innate immunity.
